# Time Rules the Efficacy of Immune Checkpoint Inhibitors in Photodynamic Therapy

**DOI:** 10.1002/advs.202200999

**Published:** 2022-04-25

**Authors:** Qinghua Wu, Yang Chen, Qing Li, Junmeng Chen, Junfeng Mo, Ming Jin, Qianzhan Yang, Loris Rizzello, Xiaohe Tian, Lei Luo

**Affiliations:** ^1^ College of Pharmaceutical Sciences Southwest University Chongqing 400715 China; ^2^ Analytical Instruments Department Analytical Applications Center Shimadzu (China) Co., Ltd. Chongqing Branch Chongqing 404100 China; ^3^ Department of Pharmaceutical Sciences University of Milan Milan 20133 Italy; ^4^ Huaxi MR Research Center (HMRRC) Department of Radiology Function & Molecular Imaging Key Lab Sichuan University Chengdu 610041 China

**Keywords:** combinational therapy, immune checkpoint inhibitors, nanomedicine, photodynamic therapy, timing

## Abstract

Lack of adequate effector T cells infiltrated in tumor is one of the main problems in the failure of immune checkpoint blockade therapy (ICBT). Photodynamic therapy (PDT) induced acute inflammation can sensitize tumors and activate T cells, thus assisting immune checkpoint inhibitors (ICI) against tumor growth and metastasis. T cells maturation and activation lag 3 to 7 days behind PDT. However, such timing in the combination therapy of ICI and PDT is commonly ignored in designing numerous multi‐functional integrated nanomedicines. Herein, the authors illustrate that intervention timing of ICI after PDT affects the anti‐tumor efficacy. A tumor‐targeting nanomedicine is prepared by encapsulating indocyanine green into CD44 specifically binding material, a hyaluronic acid conjugated lipid poly(ethylene glycol). The PDT nanomedicine is designed to induce a robust immune response in tumor. The optimal group (Combo‐STAR), ICI gave 5 days after PDT, significantly suppresses local tumor growth and eliminates metastasis. What should be highlighted is the time point of administration because if ICI is given too early, T cells are immature, otherwise, T cells are exhausted if ICI is given too late. This work presents theoretical guidance for raising awareness of intervention timing when augmenting ICBT with immune response inducers in clinic.

## Graphical abstract

Lack of adequate effector T cells infiltrated in tumor is one of the main reasons for the failure of immune checkpoint blockade therapy (ICBT). Photodynamic therapy‐induced acute inflammation can sensitize tumor and activate T cells in 3 to 7 days. This work shows that “time” is a decisive factor in the combined efficacy of photodynamic therapy (PDT) and immune checkpoint inhibitors (ICI).

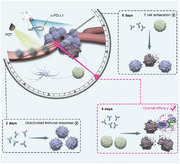

## 1 Introduction

Immunotherapy is a revolutionary treatment that boosts the host immune system to fight cancers.^[^ ^1^ ^]^ In particular, immune checkpoint blockade therapy (ICBT) such as inhibitors of the programmed cell death‐1 (PD‐1) and its major ligand the programmed cell death‐ligand 1 (PD‐L1), or inhibitors of cytotoxic T lymphocyte‐associated antigen‐4 (CTLA‐4), achieved remarkable success in the clinic.^[^ ^2^, ^3^, ^4^, ^5^ ^]^ However, in certain types of cancer, such as non‐small cell lung cancer, renal cell carcinoma, and advanced triple‐negative breast cancer, PD‐1/PD‐L1 inhibition therapy has limited help for patients.^[^ ^6^, ^7^, ^8^ ^]^

Lack of adequate effector T cells is one of the main problems for the failure of ICBT.^[^ ^9^, ^10^ ^]^ A smart strategy is to use chemotherapy, radiotherapy, or photodynamic therapy (PDT) to stimulate the generation and activation of effector T cells. Among these immune response inducers, PDT has superiority over others in terms of its non‐invasiveness and high effectiveness in generating reactive oxygen species (ROS).^[^ ^11^, ^12^, ^13^ ^]^ PDT can sensitize tumors to ICBT by inducing acute inflammation and promoting T cell activation.^[^ ^14^, ^15^, ^16^, ^17^, ^18^ ^]^ During this process, a large number of tumor cells are ablated and ruptured, leading to Toll‐like receptor activation, which triggered numerous oxidative stress signaling pathways and enhanced the expression of heat shock proteins, NF‐*κ*B and activator protein‐1.^[^ ^4^, ^19^ ^]^ These proteins can induce the expression of immunoregulatory proteins and pro‐inflammatory proteins such as interleukin (IL‐1*α*, IL‐1*β*, IL‐2, IL‐6, IL‐8, IL‐11, and IL‐12), tumor necrosis factor, chemokines and interferon (IFN‐*α*/*β*).^[^ ^20^, ^21^, ^22^ ^]^ The PDT‐induced acute inflammation will eventually lead to dendritic cells (DCs) maturation and activation, which then migrate to the tumor‐draining lymph node to activate CD4^+^ T and CD8^+^ T cells to eradicate tumor cells.^[^ ^14^, ^23^, ^24^, ^25^, ^26^, ^27^ ^]^

PDT‐induced T cell maturation requires sufficient accumulation of photosensitizer (PS) in tumor site. Micelles delivery system is an effective way to improve the stability of active pharmaceutical ingredients and increase their selective distribution in tumor site. In most of the current studies, PS and immune checkpoint inhibitors (ICI) were packed in a formulation that simultaneously targeted tumor tissue.^[^ ^28^ ^]^ However, this approach ignored two key issues: 1) Time is crucial. It is reported that approximately 3 ≈ 7 days are needed for T cells maturation upon PDT, hence dosing two agents together may miss the optimal curative opportunity.^[^ ^29^, ^30^, ^31^, ^32^ ^]^ 2) Stability of those agents needs to be guaranteed. ROS and high temperature during PDT will seriously affect the stability of *α*‐PD‐L1 when PS and ICI work at the same location and time.^[^ ^33^ ^]^

**Scheme 1:**
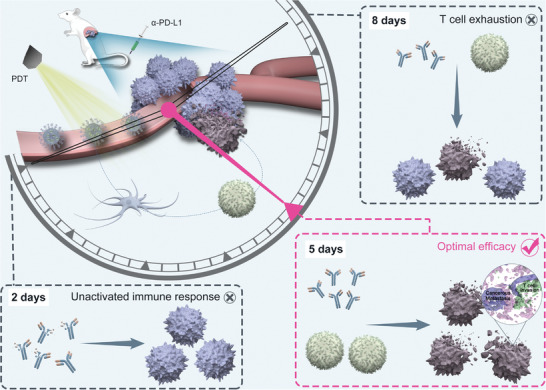
Illustration diagram of “Time” determines the combination efficacy of photodynamic therapy and immune checkpoint blockade therapy.

We herein demonstrate the importance of “Time” in the combination of PDT and *α*‐PD‐L1 (**Scheme** **1**). HIM (ICG loaded micelles with hyaluronic acid corona), is formulated with DSPE‐PEG‐HA as a delivery vehicle to encapsulate indocyanine green (ICG) in order to enhance its solubility and stability. The tumor targetability of HIM is confirmed by flow cytometry, confocal laser scanning microscope, and in vivo imaging system. The treatment time between HIM and *α*‐PD‐L1 was scheduled and a 4T1 breast cancer orthotopic model was used to evaluate the anti‐tumor and anti‐metastasis efficacy. The T cells maturation, infiltration, and exhaustion in tumor after PDT were carefully studied. Tumor cell elimination process in orthotopic and metastasis tumor under the optimized combination time were studied by 3D stimulated emission depletion assays. Our work shows that “Time” is a decisive factor in the combination of PDT and *α*‐PD‐L1, and provides an important theoretical basis for the clinical translation of PDT and ICBT.

## 2 Results and Discussion

### 2.1 Design, Preparation, and Characterization of HIM

To assess the impact of “time lag” on the combined efficacy of PDT and ICBT, a PDT nano‐agent HIM, possessing tumor‐targeting characters, was designed. Specifically, an amphiphilic polymer DSPE‐PEG‐HA was prepared by conjugating ‐COOH on HA and –NH_2_ on PEG with EDC/HOBt activation. This polymer could form micelles (HBM) in water via self‐assembly induced by hydrophobic interactions. Subsequently, the hydrophobic cores of HBM were loaded with ICG (HIM) with an encapsulation efficiency of 36.29% (Table S1, Supporting Information). The HA corona of HIM was designed for targeted delivery by specifically binding to CD44 receptors on tumor cell surface. The micelles without HA modification (IM) were prepared as control by using DSPE‐PEG‐NH_2_.

**Figure 1:**
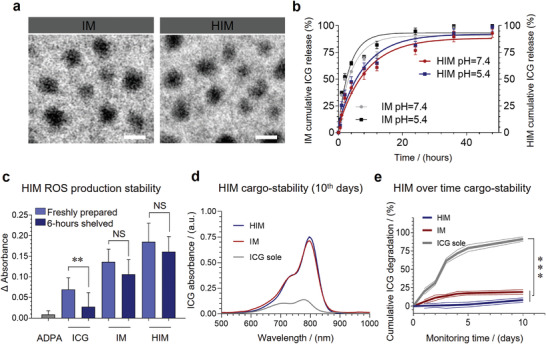
Characterization and physical stability of HIM. a) Morphologies of IM and HIM were captured by TEM. Scale bars, 50 nm. b) In vitro releasing behavior of ICG from IM and HIM under pH 7.5/pH 5.4 (*n* = 3). c) Reduction of singlet oxygen (^1^O_2_) yield after samples (*C*_ICG_ = 10 µg mL^−1^, *C*_ADPA_ = 20 µmol L^−1^) stored for 6 h (*n* = 3). Degradation rates of samples during storage (*C*_ICG_ = 10 µg mL^−1^, 25 ℃) d) on day 10 and e) within 10 days. (*n* = 3). In b, c, and e, data are presented as mean ± standard deviation (SD). For significance analysis, a two‐tailed *t*‐test (c and e) was performed. NS, no significance **p < 0.01, ***p < 0.001.

HIM showed a spherical shape with an average diameter of 41.9 nm by transmission electron microscope (TEM) imaging (**Figure** **1** **a**). The hydrodynamic diameter measured by dynamic light scattering (DLS) was increased to approximately 136.5 nm, which indicated that a large hydrophilic corona was formed (Figure S7 and Table S1, Supporting Information). ICG displayed sustained release profiles from both micelles (Figure 1b), in particular, less than 50% of ICG was released from HIM within 10 hours at pH 7.4 or pH 5.4. This might be because hydroxyl‐enriched HA corona could form a gel‐like layer with water, which could decrease the permeability.

ROS has a major contribution to PDT efficacy. The results showed that HIM generated more ROS than IM and ICG under the same condition (Figure 1c and Figure S11, Supporting Information). ICG was unstable due to self‐aggregation and irreversible degradation when stored in an aqueous solution. ROS production decreased by 46.33% after 6 h of storage. On contrary, ROS production of HIM decreased by only 12.97%. Compared with the IM group (decreased 21.96%), HIM could further improve ICG stability (Figure S8, Supporting Information). The results indicated that ICG preferably generates ROS in the oily DSPE core of this micelle instead of an aqueous solution. The ICG in HIM content was similar after 10 days, and degradation was less than 20% (Figure 1d,e and Figure S9, Supporting Information). The absolute Zeta potential value of HIM is around 40, which contributes to the stability in blood circulation through strong electrostatic action (Figure S10 and Table S1, Supporting Information). Accompanying with decent stability, HIM generated sufficient ROS to blast tumor cells and stimulate an immune response.

### 2.2 Cellular Assays and Targeting Effect

**Figure 2:**
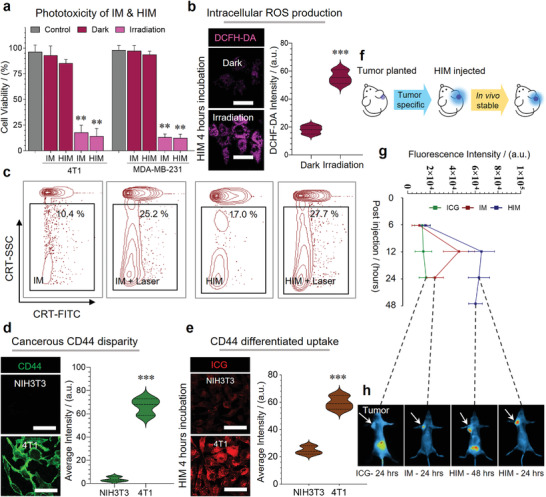
In vitro PDT efficacy and biodistribution of HIM. a) Phototoxicity of IM and HIM on 4T1 cells and MDA‐MB‐231 cells (*n* = 6). b) Intracellular ROS generation was detected by DCFH‐DA (*E*_x_ = 502 nm, *E*_m_ = 523 nm), irradiated by 808 nm laser with 372 J cm^−2^ intensity. Scale bars, 50 µm. c) Expression of calreticulin (CRT) on 4T1 cells after laser irradiation (808 nm, 60 J cm^−2^), FCM positive cells were measured. d) CD44 expression (*E*_x_ = 488 nm, *E*_m_ = 520 nm) and e) cellular uptake of HIM (*C*_ICG_ = 10 µg mL^−1^, *E*_x_ = 780 nm, *E*_m_ = 810 nm) on NIH3T3 and 4T1 cells. Scale bars, 50 µm. f) Schematic diagram of tumor modeling and HIM administration. Validation of HIM biodistribution on orthotopic 4T1 breast cancer model, g) the quantification of tumor average fluorescence intensity, and h) in vivo fluorescence images at representative time points (*E*_x_ = 780 nm, *E*_m_ = 810 nm, *n* = 3). The fluorescence intensities in b, d, e, and h were facile quantified. Significance analysis was performed using a two‐way ANOVA (a) or two‐tailed *t*‐test (b, d, and e). **p < 0.01, ***p < 0.001.

HIM significantly inhibited more than 80% viability of MDA‐MB‐231 cells and 4T1 cells with laser irradiation (**Figure** **2** **a**). Moreover, Figure S12, Supporting Information, showed that the viability of both cells decreased with increasing illumination time (2 ≈ 20 min) and intensity (0.20 ≈ 0.62 W cm^−2^). A large amount of cytotoxic ROS was detected intracellularly by irradiating the HIM treated cells (Figure 2b and Figure S13, Supporting Information).

CRT translocate from the endoplasmic reticulum to the cell surface while the PDT process, releasing a signal of “eat‐me” and binding to the receptor CD91/LRP1 on the surface of immune cells (e.g., macrophages and dendritic cells) thereby promoting T cell maturation.^[^ ^34^ ^]^ Fluorescence‐activated cell sorting results showed that HIM treated with laser led to a significant increase (27.7%) of CRT expression (Figure 2c), which was approximately 9 times higher than that of the control group (3.8%). This result was further confirmed by immunofluorescence imaging (Figure S16, Supporting Information).

Microscopy and bioluminescence systems were applied to determine the in vitro and in vivo targeting abilities of HIM, respectively. HIM was able to effectively distinguish cancer from noncancerous cells, due to differential expression of CD44 on cell membrane (Figure 2d,e and Figure S14, Supporting Information). Furthermore, the in vivo targeting efficacy of HIM was evaluated on an orthotopic breast cancer mice model (Figure 2f). HIM was accumulated in tumors after 24 h and then encapsulated ICG was released and absorbed, finally discharged into the intestine through bile and excreted with feces. The maximal retention of ICG occurred approximately 12 h after i.v. injection (Figure 2g,h), indicating the proper laser irradiation timing. On the contrary, the biodistribution of free ICG showed non‐specificity and was quickly eliminated by the liver. (Figure S15, Supporting Information). These properties guarantee it as a promising PDT tool in the following study.

### 2.3 In vivo Anti‐Tumor and Anti‐Metastatic Efficacy

**Figure 3:**
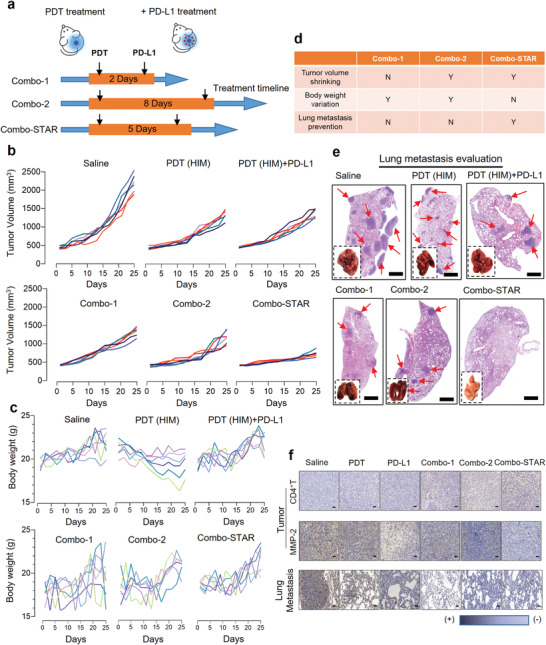
In vivo anti‐tumor and anti‐metastatic efficacy studies. a) Dosage regimen illustration. b) Evaluation of in vivo anti‐tumor efficacy by tumor volume on orthotopic breast cancer mice model (*n* = 6). c) Body weight variation within 25 days (*n* = 6). d) Overall efficacy and safety evaluation of Combo‐plans. e) Appearance of the dissected lung and H&E staining of pulmonary sections after treatments (red arrows point to metastatic nodes. Scale bars, 1000 µm). f) Immunohistochemistry for CD4^+^ T, MMP‐2 in tumors and lungs. Scale bars, 50 µm.

As schematically illustrated in **Figure** **3** **a**, the dosage intervals on the orthotopic cancer model were set as 0, 2, 5, and 8 days between PDT and ICI. The tumor volume and body weights were recorded for 25 days (Figure 3b,c). The overall efficacy and safety evaluation of Combo strategies is shown in Figure 3d. It was observed that the Combo‐STAR group significantly inhibited tumor growth with an impressive inhibition rate of 75.99%. By comparing with the efficacy of the other groups, the results indicated that time played a crucial role in the combination of PDT and ICI (Figure S17 and Table S2, Supporting Information). This may be largely related to the degree of T cell infiltration at the tumor site during the intervention of immune checkpoint inhibitors.^[^ ^35^, ^36^ ^]^ In the cases of HIM+PD‐L1 and Combo‐1 groups, PD‐L1 is difficult to play a practical role due to a lack of matured T cells, while in the Combo‐2 group T cells were exhausted. In these cases, PDT likely dominates the anti‐tumor treatments thus these groups showed similar efficacy. Moreover, the Combo‐STAR treatment was able to inhibit breast cancer cell metastases to the lung (Figure 3e). Although Combo‐1 and Combo‐2 groups showed a certain degree of effects, obvious metastasis was observed from the lung section (Figure S18, Supporting Information). The tumor section of the Combo‐STAR group showed minimal expression of cancer metastasis biomarkers, such as Ki67, MMP‐2, and MMP‐9.^[^ ^37^ ^]^ These biomarkers and metastatic nodules were also not observed in the pulmonary region in the Combo‐STAR group (Figure 3f and Figure S19 Supporting Information).^[^ ^38^ ^]^ The prevention of metastasis is critical from a clinical point of view, since the metastatic cancer cells might lead to tumor recurrence and relapse.^[^ ^39^, ^40^, ^41^ ^]^

In addition, the secretion of cytokines such as IFN‐*γ*, IL‐6, and TNF‐*α* in tumors after PDT also confirmed the activation of T cells (Figure S21, Supporting Information). And the tumor section of the Combo‐STAR group showed an optimal infiltration of CD4^+^ T and CD8^+^ T cells (Figure 3f first line and Figure S20, Supporting Information). The same conclusion can be drawn from Figure S22, Supporting Information, a large number of CD4^+^ T cells and CD8^+^ T cells were infiltrated in the tumor sites of Combo‐groups, especially the Combo‐STAR group showing the highest number of activated T cells. An adequate amount of intratumoral CD4^+^ T and CD8^+^ T cells is an essential requirement for effective *α*‐PD‐L1 work.

### 2.4 Immunological Responses During Combo‐STAR Treatment

**Figure 4:**
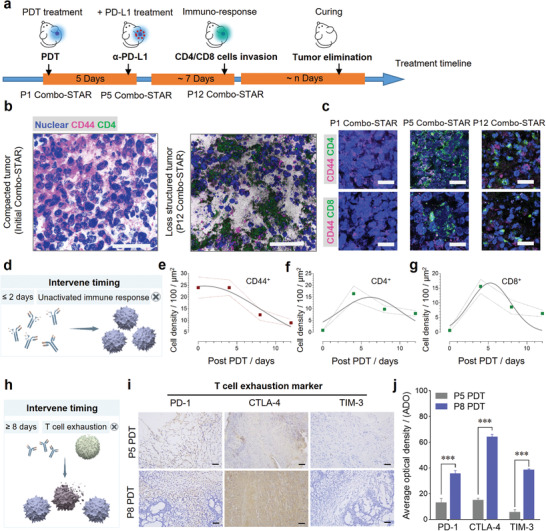
T cells infiltration and exhaustion at tumor site during treatment. a) Schematic illustration of Combo‐STAR treatment timeline. b) Tumor cells were eliminated by T cells in the therapeutic process on the 1^st^ and 12^th^ days. Scale bars, 50 µm. c) Concomitant, intratumoral CD4^+^ T and CD8^+^ T cells infiltration in the therapeutic process on the 1^st^, 5^th^, and 12^th^ days. Scale bars, 50 µm. d) Immature immune response during the premature intervention. Chronological variation in density of e) CD44^+^ tumor cells, f) CD4^+^ T and g) CD8^+^ T cells in tumor sections over 12 days. Each mean number of cells was calculated from 9 equal regions (50 µm × 50 µm). h) Exhausted T cells with extended intervening timing. i) Immunohistochemistry staining and j) average optical density (ADO) of T cell exhaustion markers PD‐1, CTLA‐4, and TIM‐3 on tumor slices at the 5^th^ and 8^th^ days (Scale bars, 50 µm). ADO was acquired by the average gray value of positive cells/positive area, and each mean value was calculated from 6 equal regions (100 µm × 60 µm). For significance analysis, a two‐tailed *t*‐test (j) was performed. ***p < 0.001.

The decent efficacy of “Time” dependent combination therapy motivated us to further investigate T cell infiltration with “too short” and “too long” intervention timing of ICI (**Figure** **4** **a**). The tumors were harvested on the 1^st^ and 12^th^ days after PDT, respectively (Figure 4b). The results showed that quantities of tumor cells were eliminated on day 12 and CD44, a biomarker of tumor cells, signals were almost lost. The intratumoral CD4^+^ T and CD8^+^ T cells within 12 days were also tracked. The amount of both T cells increased progressively with time, culminating around the 5^th^ day, followed by a gradual decline (Figure 4c). It suggested that “too short” the intervene timing, due to the lack of sufficient activated T cells in the tumor, is limiting ICI to exert its effect (Figure 4d). The changes of CD44, CD4^+^, and CD8^+^ fluorescence intensity in the tumor site within 12 days further demonstrated the T cell infiltration process of Combo‐STAR (Figure 4e–g). The FACS results showed a similar trend of T cell changes to the immunofluorescence results. The number of activated T cells infiltrating in tumor site increased dramatically after PDT treatment, especially on day 5. And over time, the number of activated T cells significantly decreased because of the immunosuppressive microenvironment at the tumor site (Figure S23, Supporting Information). In the initial 5 days after PDT treatment, CD4^+^ T and CD8^+^ T cells increased synchronously, and the ratio of the two was maintained in the range of 0.8–1.2, which is a representative level of T lymphocytes in response to malignant tumors.^[^ ^42^, ^43^ ^]^ With the progress of the treatment to the 6^th^ to 12^th^ day, the ratio gradually tended to the normal value (*r* > 1.4), indicating that the anti‐tumor effects have been achieved. These results showed that day 5 is the optimal timing for the best efficacy of Combo‐plans.^[^ ^44^ ^]^

T cell exhaustion is a dysfunctional state in which T cells gradually lose their effector functions due to a prolonged fight against persistent antigens and an immunosuppressive tumor environment.^[^ ^45^, ^46^ ^]^ The antigens could be generated from the tumor itself. Meanwhile, the tumor microenvironment is also enriched with immunosuppressive‐related inhibitory factors. Both factors jointly create an immunosuppressive tumor environment and continuously suppress the immune function of T cells.^[^ ^47^, ^48^, ^49^ ^]^ Therefore, “too long” the intervention timing may result in loss of effector function due to T cell exhaustion (Figure 4h). T cell exhaustion assays were performed to evaluate the effector function of T cells on tumor site on the 5^th^ and 8^th^ days. Tumor‐infiltrating CD4^+^ lymphocytes appeared largely exhausting on day 8, and a significant increase in the expression of PD‐1, CTLA‐4, and TIM‐3 was observed (Figure 4i,j).

### 2.5 3D Stimulated Emission Depletion (STED) Images Visualize T Cells Taking Effect

**Figure 5:**
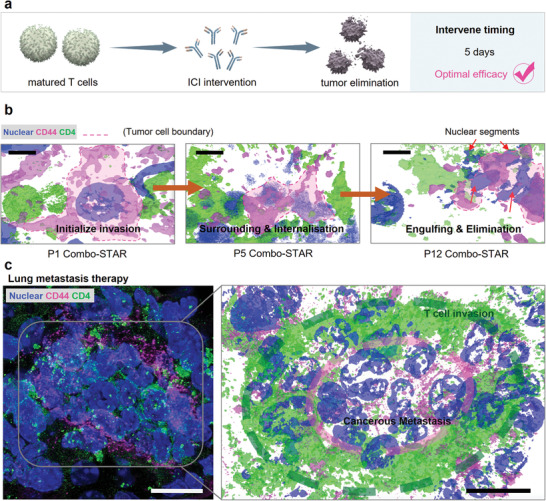
Matured T cells against orthotopic and metastasis tumor. a) The optimal anti‐tumor efficacy in Combo‐STAR. b) Visualization of Combo‐STAR enhanced immune blockade efficacy. Scale bars, 10 µm. c) T cells infiltrated (green) and sieged cancerous metastasis (pink) in the pulmonary region. Scale bars, 50 µm.

The sufficient number of activated T cells supported the efficacy of ICI, thus achieving the optimal anti‐tumor and anti‐metastatic efficacy (**Figure** **5** **a**). The interaction between T cells and tumor cells was visualized by super‐resolution STED imaging. The process of Combo‐STAR enhanced immune blockade efficacy in tumor was clearly observed as three stages: initial T cells invasion; surrounding and internalization; engulfing and eliminating (Figure 5b). The thorough intratumoral destruction can prevent tumor recurrence and metastasis. Notably, a large number of T cells invaded and sieged cancerous metastasis in the pulmonary region, which might be an explanation for immune therapy to prevent tumor cells from escaping (Figure 5c).

## 3 Conclusions

In conclusion, we illustrated the decisive role of “time” in the combination of PDT and immunotherapy. First, we established a tumor‐targeting nanomedicine which improved ICG stability and ROS production to ensure that the PDT can induce a sufficient immune response. Second, we revealed that the “intervention timing” of 5 days while dosing ICI after PDT could significantly promote the anti‐tumor and anti‐metastasis efficacy. It was further found that the tumor infiltrated T cells were immature if the intervention was premature and they were exhausted when the treatment lasted more than 8 days. It is known that immune‐therapy fails owing to comprehensive factors, our findings are expected to raise the awareness of intervention timing when promoting ICBT with immune response inducers in the clinic.

Additionally, in this study, we enabled immune checkpoint inhibitors to act on immune “cold” tumors such as the 4T1 cell xenograft model by increasing T cell infiltration. This may provide a possible alternative when clinical treatment of immunologically “cold” tumors fails. We are also investigating the “time” related immunological studies on other tumor models in further research, including “hot” tumor models. We expect to make it more widely applicable in the clinic.

## 4 Experimental Section

### Materials and Reagents

Hyaluronic Acid (HA) was purchased from Haihua (Jiangsu, China). Indocyanine green (ICG) was purchased from Aladdin (Beijing, China). DSPE‐PEG‐NH2 was supplied by Aiweituo (Shanghai, China). 1‐(3‐dimethylaminopropyl)‐3‐ethylcarbodiimide hydrochloride (EDC) was purchased from Huacheng (Tokyo, Japan). 1‐hydroxybenzotriazole (HOBt) was purchased from Dingguo Changsheng (Beijing, China). CD4 antibody, CD8 antibody, and CD44 antibody were purchased from Abcam (Cambridge, UK). Anthracene‐9, 10‐dipropionic acid disodium salt (ADPA) was purchased from Santa Cruz Biotechnology (California, USA). 2′,7′‐Dichlorodihydrofluorescein diacetate (DCFH‐DA) was purchased from Sigma‐Aldrich (St. Louis, USA). Trypsin‐EDTA, 3‐(4, 5‐dimethylthiazole‐2‐yl)‐2, 5‐diphenyltetrazolium bromide (MTT) and 4, 6‐diamino‐2‐phenylindole dihydrochloride (DAPI) were purchased from Dingguo Changsheng (Beijing, China). Fetal bovine serum (FBS) was purchased from Tianhang Biotechnology (Hangzhou, China). Triton X‐100 was purchased from Aladdin (Beijing, China). Anti‐PD1 antibody and anti‐TIM 3 antibody were purchased from Abcam (Cambridge, UK). Anti‐CTLA4 antibody were purchased from GeneTex (California, USA). All the reagents, except that were not pointed out specially, were analytical grade.

### Characterization and Apparatus

Size distribution and Zeta potential were achieved using a Zeta‐sizer (Malvern Instrument, UK). The ultraviolet and visible spectrophotometry (UV‐vis) spectra were conducted on a UV‐6100 spectrophotometer (Shanghai Mapada, China). The critical micelle concentration (CMC) was measured with F‐7000 fluorescence spectrophotometer (Hitachi, Japan). The contents of the samples were determined by HPLC (SHIMADZU, Japan). JEM‐1200EX transmission electron microscope (JEOL, Japan) was used for morphology analysis and a confocal laser scanning microscope (CARL ZEISS, Germany) for imaging and photography. The optical power is provided by an 808 nm laser (Xian Zhongchuan, China). The fluorescence absorbance value in the MTT experiment was obtained by microplate reader ELx‐800 (BioTek, USA). Drug targeting was studied by an IVIS spectrum in vivo imaging system (PerkinElmer, USA). The tissue sections were prepared using a RM 2016 rotary slicer (Leica, Germany).

### Cell Lines and Animals

Human mammary carcinoma MDA‐MB‐231 cell line, mouse breast cancer 4T1 cell line, and mouse embryonic fibroblasts NIH3T3 cell line were purchased from Nanjing Kaiji Co., Ltd. Cells were cultured in DMEM medium containing 10% fetal bovine serum in a humidified incubator with a 95% air / 5% CO_2_ atmosphere at 37 °C.

SPF female BALB/c mice (18 ± 2 g, 5–7 weeks) were purchased from Beijing huafukang Co., Ltd., and were bred in a room under specific pathogen‐free conditions with a 12 h light/12 h dark cycle. Animal experiments meet all relevant ethical regulations for animal experiments and research, and have been approved by the Institutional Animal Care and Use Committee of Southwest University (IACUC‐20200215‐01).

### Hyaluronic Acid Decoration of DSPE‐PEG

The amphiphilic copolymer DSPE‐PEG‐NH_2_ was reacted with the hydroxyl groups of HA. The detailed synthetic methods and procedures were described in the Supporting Information. The ^1^H nucleic magnetic resonance (NMR) spectra data of polymer DSPE‐PEG‐HA were obtained via a 400 MHz Bruker Advanced Spectrometer (BRUKER, Switzerland), and chemical shifts were reported in ppm on the *δ* scale.

### Preparation and Characterization of HIM

The drug‐loaded micelles with HA decoration (HIM) were prepared by an organic solvent evaporation method. In brief, ICG in methanol was added into the DSPE‐PEG‐HA aqueous solution (ICG: DSPE‐PEG‐HA = 1: 10, m/m). The mixture was stirred overnight in the ventilation cabinet away from light to remove organic reagents. Following, ultrasonication (200 W) with a pulse duration of 5 s and a resting interval of 5 s were done for the mixture over 15 min on an ice bath to avoid overly high temperatures. The final drug‐loaded micelle HIM was obtained by dialysis against purified water for 30 min and filtered by 0.22 µm pore‐sized syringe filters. The drug‐loaded micelles (IM) without HA modification were prepared with DSPE‐PEG‐NH_2_ in a similar manner. The preparation of HBM and BM also used the same method without adding ICG.

The particle size distribution and zeta potential of BM, HBM, IM, and HIM were measured with a dynamic light scattering (DLS) analyzer (the polymer mass concentration was about 1 mg mL^−1^). Measurements were conducted three times in parallel at a scattering angle of 90° to the incident beam at 25 °C. The morphologies of BM, HBM, IM, and HIM were observed under transmission electron microscopy (TEM).

A UV‐vis spectrophotometer was used to scan the absorbance of free ICG, IM, and HIM at a wavelength of 500–1000 nm, and the UV absorption spectra were recorded. Similarly, their emission spectra at 780 nm excitation wavelength were monitored by a fluorescence spectrophotometer. The encapsulation efficacy, *E*_E_, and drug loading, *D*_L_, were calculated as the following Equation (1):

(1) $$E_{E}\%=\frac{W_{L}}{W_{0}}\times 100\%,\;D_{L}\%=\frac{W_{L}}{W_{0}+W_{M}}\times 100\%$$

where *W*_L_ was the weight of the loaded drug, *W*_0_ the initial drug weight, and *W*_M_ the weight of the micelle polymer.

### Stability of Drug‐Loaded Micelles

The stability of IM and HIM at 4 °C and 37 °C was evaluated by measuring their average sizes, which were determined by Zetasizer.

Free ICG, IM, and HIM solutions containing 10 µg mL^−1^ of ICG were monitored via UV absorption spectrum within 10 days.

### In vitro Release Profiles

The dialysis method was used to investigate the release profiles of ICG from HIM and IM, respectively. The micellar dispersions were placed into a dialysis membrane (MWCO = 3500 Da) and immersed into 250 mL PBS (pH 5.4, pH 7.4) at 37 ℃ on a shaker at 100 rpm. A small volume was withdrawn from the different buffered media at a predetermined time within 48 hours, and the concentration of the released ICG was determined using UV (*λ*_max_ = 779 nm).

### Determination of ^1^O_2_

The yield of ^1^O_2_ could be determined by the decrease of absorbance at 400 nm of athracene‐9, 10‐dipropionic acid disodium salt (ADPA) due to associated bleaching characteristics of ^1^O_2_. Briefly, free ICG, IM, and HIM solutions with ICG concentration of 10 µg mL^−1^ and ADPA concentration of 20 µmol L^−1^ were prepared separately, and 20 µmol L^−1^ ADPA solution alone was used as control. The freshly prepared solution and the solution stored for 6 hours were put into a 1 cm × 1 cm × 0.5 cm quartz trough, and individually irradiated by laser (808 nm, 1080 J cm^−2^), and then the absorbance of ADPA within 1 hour at 400 nm was determined.

### Cell Viability Assays

Dark cytotoxicity and photo‐cytotoxicity of IM and HIM on 4T1 cells and MDA‐MB‐231 cells were investigated by MTT assay.

The cells were seeded in 96‐well plates and cultured overnight (4T1 cells 5 × 10^3^ per well, MDA‐MB‐231 cells 1 × 10^4^ per well). For dark cytotoxicity assays, cells were respectively treated with different concentrations of IM or HIM for 24 h avoiding light. Then, the medium was discarded and the cells were incubated for 4 h with fresh medium containing 10% MTT. Finally, MTT media were aspirated from each well and 100 µL DMSO was added to dissolve the formed formazan crystals and the absorbance was subsequently measured at 490 nm by a microplate reader (calibrated at 630nm). In terms of photo‐cytotoxicity assessment, after respectively incubated with IM or HIM for 6 h, the cells were irradiated by a laser with 808 nm, 744 J cm^−2^ for 20 min. The rest steps of MTT assays were performed to determine the cell viability. In all cases, the blank value measured in the wells containing DMSO and cell‐free was subtracted. The cell viability was calculated according to the following formulation: cell viability (%)  =  OD experiment/OD control × 100%.

### Confocal Microscopy and Imaging

Cells were inoculated in a confocal culture dish and allowed to attach overnight. For intracellular ROS detection, MDA‐MB‐231 cells were incubated with a medium containing IM or HIM (*C*_ICG_ = 10 µg mL^−1^) for 4 h, washed with PBS three times, and then incubated with DCFH‐DA for an additional 20 mins before being irradiated with a laser (808 nm, 372 J cm^−2^). Subsequently, the fluorescence signal of DCFH‐DA was detected by confocal microscopy (*E_x_* = 502 nm, *E*_m_ = 523 nm). For the CD44 receptor expression assay, 10 µL of CD44 monoclonal antibody labeled with FITC was added into the DMEM medium of 4T1 cells and NIH3T3 cells under dark conditions and incubated for 1 h at 37 ℃ in 95% air and 5% CO_2_. Images were acquired under laser scanning confocal microscopy (*E_x_* = 488 nm, *E_m_* = 520 nm). For cell uptake imaging, 4T1 and NIH3T3 cells were maintained at 37 °C and 5% CO_2_ in the medium containing ICG, IM, or HIM (*C*_ICG_ = 10 µg mL^−1^) for 4 h. Then, the cells were washed with PBS three times and observed by laser scanning confocal microscopy (*E_x_* = 780 nm, *E*_m_ = 810 nm) to detect the concentration of ICG in the cells. 100 cells were encircled under brightfield conditions for quantification, which was performed via ImageJ software.

### Detection of CRT

The expression of CRT was detected by immunofluorescence and flow cytometry. Briefly, 4T1 cells were plated in 6‐well culture plates overnight, and incubated with free ICG, IM, and HIM (C_ICG_ = 10 µg mL^−1^) for 6 h, respectively. Then, the drug‐containing medium was replaced by a 100 µL fresh medium. Cells were subsequently irradiated with a laser (808 nm, 60 J cm^−2^), fixed in 4% paraformaldehyde for 30 min, and incubated with 5% BSA for 2 h. Cells were stained with the primary antibody: Anti‐Calreticulin antibody‐ER Marker (1:100) at 4 °C overnight, and subsequently with the secondary antibody: Dylight 488 Goat Anti‐Rabbit IgG (1:1200) at 37 ℃ for 1 h. Finally, cells were co‐stained with DAPI for nuclei visualization and observed by fluorescence microscope. All steps were completed in dark conditions.

In flow cytometry assay, 4T1 cells were seeded to a 6‐well plate (5 × 10^6^ per well) overnight. The DMEM medium containing IM or HIM (*C*_ICG_ = 10 µg mL^−1^) was added to treat for 6 hours, and the experiment was designed in **Table** **1**.

**Table 1:** FACS experiments design

	1	2	3	4	5	6	7	8
Treatment	Medium	Medium	ICG	ICG	IM	IM	HIM	HIM
Irradiate	−	+	−	+	−	+	−	+

※ the dosage was 10 µg mL^−1^ according to ICG.

+ indicates the wells with laser irradiation (808 nm, 345.6 J cm^−2^).

− without laser irradiation.

After treatment, cells were harvested, washed with PBS, and stained. The expression of CRT was examined using flow cytometric measurement and assayed by Flowjo software.

### Orthotopic Breast Cancer Mice Model

After a 1‐week adaptation period, an orthotopic breast cancer tumor model was established by inoculating 0.1 mL suspension of 4T1 murine breast cancer cells (5 × 10^5^) into the mammary fat pad of Balb/C mice.

### In Vivo Fluorescence Imaging

4T1 tumor‐bearing mice were randomly divided into three groups (*n* = 3 in each group) when the tumor volume reached 150–200 mm^3^. The mice were injected with ICG, IM, and HIM solution (*C*_ICG_ = 6 mg kg^−1^) individually via the caudal vein. Mice were fed in the dark after administration. Then, the bioluminescence images were acquired at 0, 6, 12, 24, 36, and 48 h by the IVIS spectrum in vivo imaging system. The fluorescence intensity was quantified in ImageJ software, the bright field delineation method was used for shown tumor area, and same size of area on the opposite side was selected as the background.

### In Vivo Anti‐Tumor and Anti‐Metastasis Efficacy Assays

The model mice were randomly divided into six groups (*n* = 6) when the tumor volume reached 100 mm^3^, and the experiment was designed in **Table** **2**.

**Table 2:** In vivo anti‐tumor therapeutic regimens

Treatment Groups	Saline	PDT	PD‐L1	Combo‐I	Combo‐II	Combo‐STAR
Tail vein	PBS	HIM	PBS	HIM	HIM	HIM
Intraperitoneal	PBS	PBS	*α*‐PD‐L1	*α*‐PD‐L1*2	*α*‐PD‐L1*5	*α*‐PD‐L1*8
Laser	−	+	−	+	+	+

※ the dosage was 10 µg mL^−1^ according to ICG, and the dosage of *α*‐PD‐L1 was 100 µg per mouse.

+ indicates the wells with laser irradiation (808 nm, 345.6 J cm^−2^).

− without laser irradiation.

*2 means *α*‐PD‐L1 was injected 2 days after PDT

*5 means *α*‐PD‐L1 was injected 5 days after PDT

*8 means *α*‐PD‐L1 was injected 8 days after PDT.

All treatments were performed one time in total. The tumor volume (length × width^2^ × 0.5) and body weight were measured every 2 days during the 25 day treatment period. All mice were sacrificed on day 25 due to the fatal nature of this model. The tumor and lung were dissected for histopathological examination and immunohistochemistry analysis, while the excised tumors were photographed and weighed additionally. Heart, livers, spleens, and kidneys were also excised for histopathological analysis.

### Immunofluorescence and Immunohistochemistry

Tumors of the Combined‐STAR group were collected, fixed in 4% paraformaldehyde, and embedded in paraffin. The paraffin sections with 5 µm thickness were prepared using a LEICA rotary slicer, followed by deparaffinization and hydration, after which immunofluorescence and immunohistochemistry assay was performed.

The sections were boiled in a sodium citrate buffer (pH 6.0) for 30 mins to retrieve antigens, subsequently permeabilized with 0.5% Triton X‐100 in PBS for 5 mins, incubated with 100 mM glycine for 15 mins, and blocked with 1% BSA for 1 h at room temperature. Primary antibodies for immunofluorescence were included anti‐CD4, rabbit, 1:1000, anti‐CD8 alpha, rabbit, 1:2000, and anti‐CD44, rat, 1:250. Primary antibodies for immunohistochemistry were included anti‐PD1, mouse, 1:50, anti‐CTLA4, rabbit, 1:100, anti‐TIM3, rabbit, 1:1000. Secondary antibodies include goat anti‐rabbit 647 (*E_x_* = 495 nm, *E*_m_ = 519 nm) and goat anti‐rat FITC (*E*_x_ = 652 nm, *E*_m_ = 668 nm). DAPI was used for nuclear staining prior visualized by laser scanning confocal microscopy, and the intensity of immunohistochemistry positive staining was measured by ImageJ software.

### Statistical Analysis

All experiments were repeated at least three times. The results were expressed as mean ± SD. Results were analyzed by two‐way ANOVA with a multiple‐comparison test for multiple groups and a two‐tailed *t*‐test for two groups. All statistical analyses were performed using Graphpad Prism 8 (Version 8.0.1). *p < 0.05, **p < 0.01 and ***p < 0.001 were considered statistically significant.

## Author Contributions

L. Luo, Q. Wu, and Y. Chen conceived this project. Q. Wu and Y. Chen carried out the experiments and analyzed the results. L. Luo, Q. Wu, and X. Tian drafted the manuscript. L. Luo, X. Tian, L. Rizzello, Q. Li, J. Chen, J. Mo, M. Jin, and Q. Yang critically revised the manuscript. All authors approved the final version of the manuscript.

## Conflict of Interest

The authors declare no conflict of interest.

## Supporting information

Supporting Information

## Data Availability

The data that support the findings of this study are available from the corresponding author upon reasonable request.
