# Supplementary material for: Time Rules the Efficacy of Immune Checkpoint Inhibitors in Photodynamic Therapy
Source: Adv Sci (Weinh). 2022 Apr 25;9(21):2200999. doi: 10.1002/advs.202200999 (PMC9313507; doi:10.1002/advs.202200999)
Supplement: Supplementary file 1 — Supporting Information [file ADVS-9-2200999-s001.pdf]

ADVANCED SCIENCE
Open Access

Supporting Information

for *Adv. Sci.*, DOI 10.1002/advs.202200999

Time Rules the Efficacy of Immune Checkpoint Inhibitors in Photodynamic Therapy

*Qinghua Wu, Yang Chen, Qing Li, Junmeng Chen, Junfeng Mo, Ming Jin, Qianzhan Yang, Loris Rizzello, Xiaohe Tian and Lei Luo**

Supporting Information

Time rules the efficacy of immune checkpoint inhibitors in photodynamic therapy

*Qinghua Wu, Yang Chen, Qing Li, Junmeng Chen, Junfeng Mo, Ming Jin, Qianzhan Yang, Loris Rizzello, Xiaohe Tian, Lei Luo**

Q. Wu, Y. Chen, Q. Li, J. Chen, J. Mo, M. Jin, L. Luo

College of Pharmaceutical Sciences

Southwest University

Chongqing 400715, China

E–mail: drluolei@swu.edu.cn

Q. Yang

Analytical Instruments Department, Analytical Applications Center

Shimadzu (China) Co., Ltd.

Chongqing 404100, China

L. Rizzello

Department of Pharmaceutical Sciences

University of Milan

Milan 20133, Italy

X. Tian

Huaxi MR Research Center (HMRRC), Department of Radiology, Function & Molecular Imaging Key Lab

Sichuan University

Chengdu, 610041 China

Synthesis and characterization of DSPE-PEG-HA

Following the synthesis route shown in Supplementary Fig. 1, HA (20 μmol) and EDC (5 μmol) were stirred and reacted in PBS solution (pH = 6.8) for 1 hour, then the methanol solution of HOBt (5 μmol) was dropwisely added to activate the carboxyl group on HA for different times (4, 10, 24 or 48 h), and then the PBS solution with a total of 10 μmol of DSPE-PEG-NH_2 was slowly added into the mixed solution, the polymerization was quenched 24 hours later. The resulting solution molecules were dialyzed with excess methanol for 24 hours through a dialysis bag with an MW cut-off of 7000 DA, followed by pure water : methanol (V : V) = 1 : 3 for one day and pure water: methanol (V : V) = 1 : 1 for another day. After lyophilization, the final product DSPE-PEG-HA was obtained as a white powder. ^{1}H NMR spectroscopy data of polymer DSPE-PEG-HA were obtained via a 400 MHz Bruker Advanced Spectrometer (BRUKER, Switzerland), and the chemical shifts were reported in ppm on the δ scale.

With the prolonging of activation time of carboxyl group, the yield also increased, but the water-solubility of the polymer also decreased gradually. As shown in Supplementary Fig. 2, the peak pattern of the chemical shift value of the polymer at 4.52 ppm becomes sharper and sharper with the increase of activation time, which was probably due to the increasing number of DSPE-PEG-NH_2 bonded on HA molecule leads to poor water-solubility of the product, resulting in a stronger and sharper peak of solvent D_2O. After comprehensive consideration, we chose 10 hours as the optimal activation time of carboxyl for subsequent experiments. The yield of DSPE-PEG-HA was 36.29%. The ^{1}H NMR spectroscopy data shown in Supplementary Fig. 3 showed the substitution degree of HA on polymer was 15.80 %.

Determination of critical micelle concentration (CMC)

The critical micelle concentration (CMC) of DSPE-PEG-HA was estimated using fluorescence spectroscopy with pyrene as a probe. 1 mL of pyrene solution in acetone (5×10^{-6} mol/L) in a brown bottle was stirred in a fume hood in dark until the acetone evaporated. DSPE-PEG-HA solution diluted with PBS at different concentrations ranging from 0.01 μg/mL to 1.0 mg/mL was added to each bottle, and

the solution was stirred for 24 h in a constant temperature magnetic stirrer. The emission spectra of pyrene were monitored by a fluorescence spectrophotometer (E_m=334 nm) with an excitation wavelength of 334 nm and a slit width of 5 nm. The intensity ratios of I_{373}/I_{384} were plotted against the DSPE-PEG-HA concentration to determine CMC. The CMC of DSPE-PEG-NH_2 was estimated by the same method and the result was shown in Supplementary Fig. 4.

Preliminary characterization of drug-loaded micelles

As shown in Supplementary Fig. 5, an ultraviolet-visible (UV) spectrophotometer was used to scan the absorbance of free ICG, IM, and HIM at a wavelength of 500-1000 nm and the UV absorption spectra were recorded. Similarly, their emission spectra at 780 nm excitation wavelength were monitored by a fluorescence spectrophotometer.

The standard curve of ICG aqueous solution at 779nm was determined by UV spectrophotometer (Supplementary Fig. 6). The regression equation was y = 0.2056x + 0.0661, R^2 = 0.9991, indicating that ICG had a good linearity in the concentration range of 1-5 μg/mL.

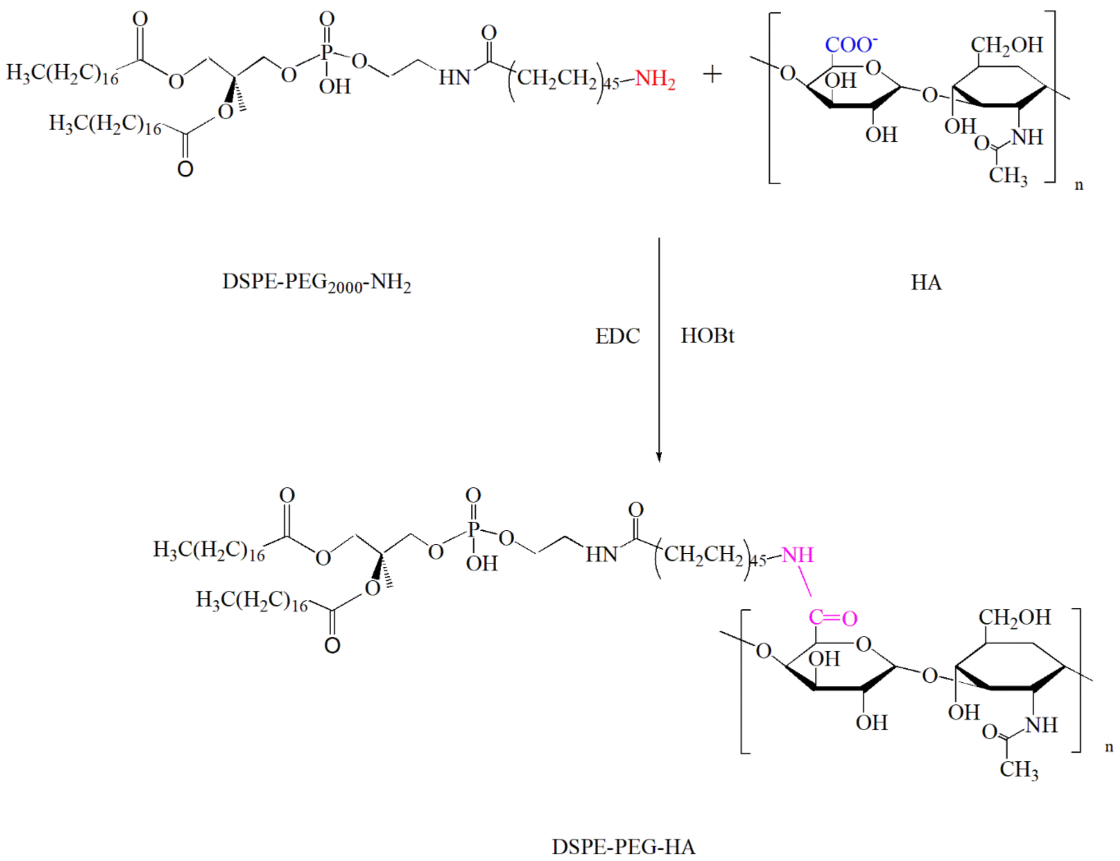

Supplementary Fig. 1. Synthesis diagram of DSPE-PEG-HA.

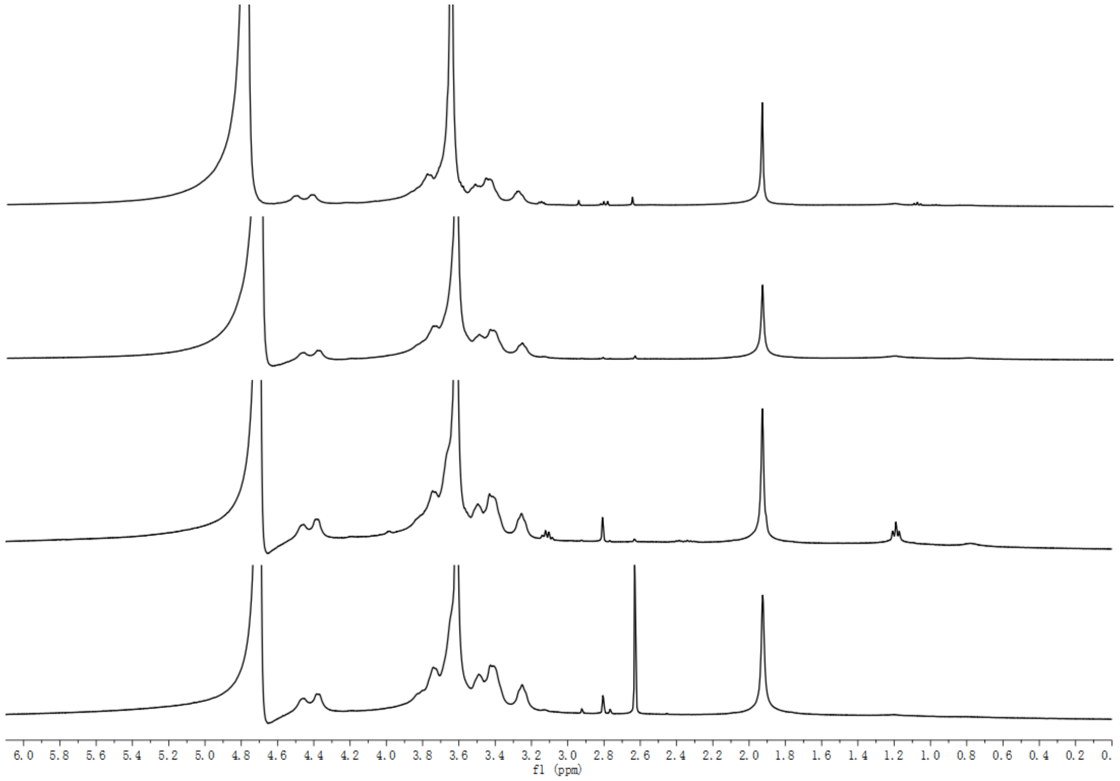

Supplementary Fig. 2. ^{1}H NMR spectra of DSPE-PEG-HA with different carboxyl activation times.

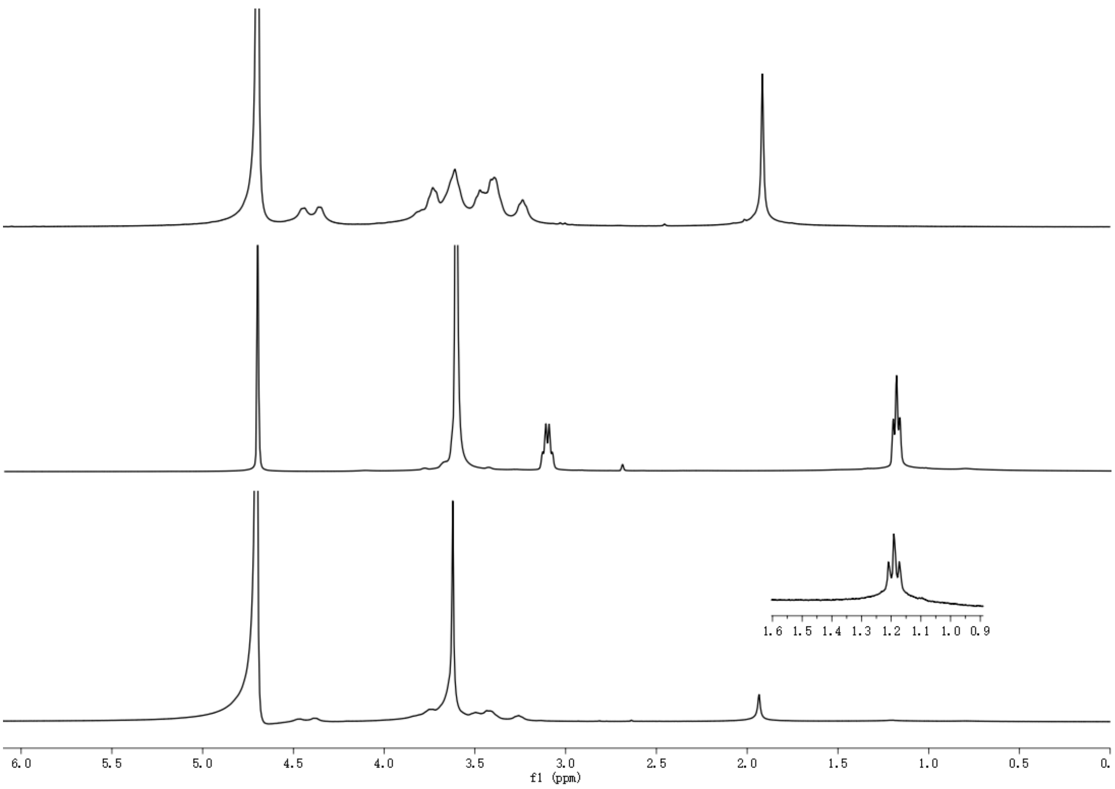

Supplementary Fig. 3. ^{1}H NMR spectra of HA, DSPE-PEG and DSPE-PEG-HA.

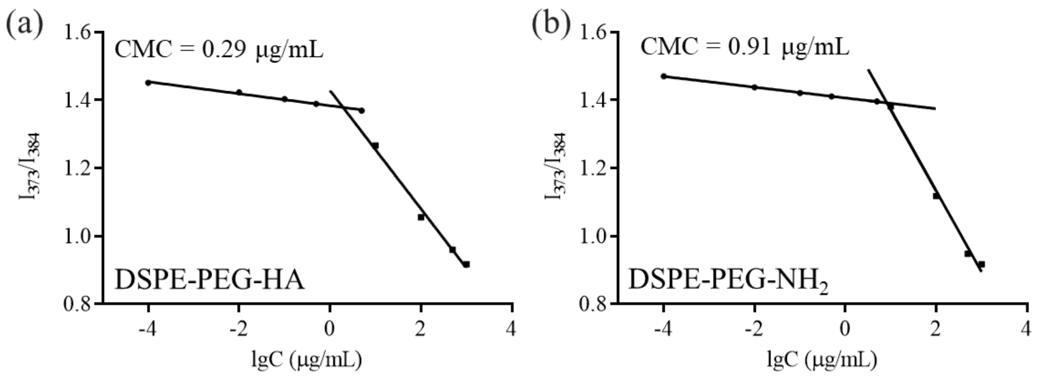

Supplementary Fig. 4. The fluorescence ratio (I_{373}/I_{384}) of pyrene to determine the CMC value of (a) DSPE-PEG-HA and (b) DSPE-PEG-NH_2.

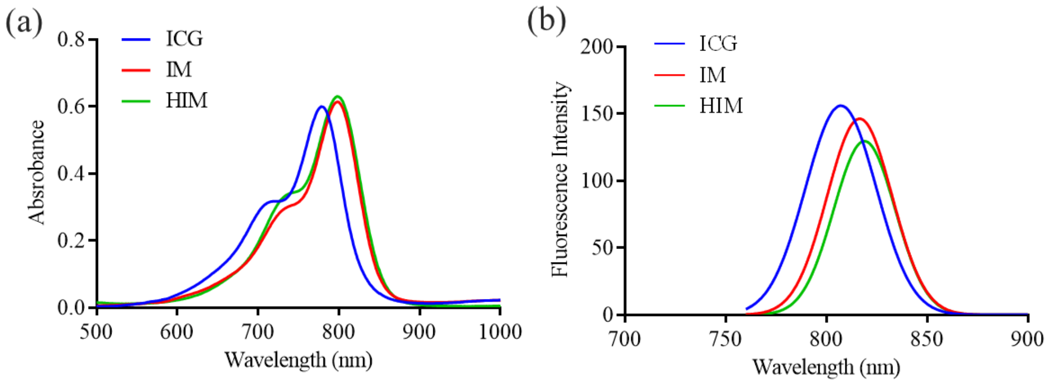

Supplementary Fig. 5. (a) UV-Vis spectra and (b) fluorescence spectra of ICG, IM and HIM (E_x=780 nm).

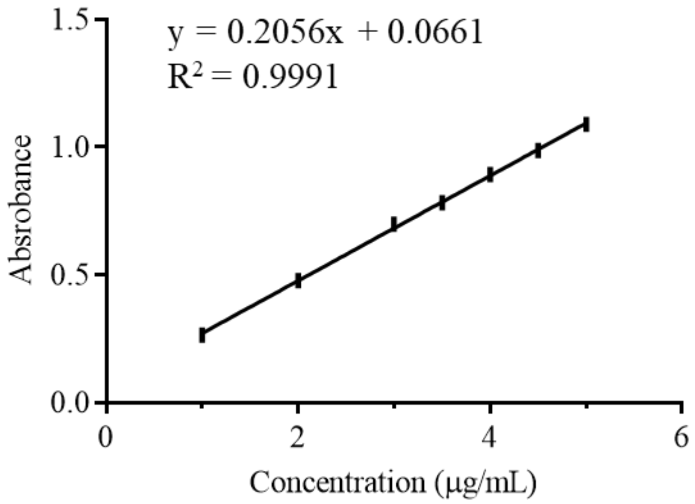

Supplementary Fig. 6. Standard curve of ICG in deionized water at 779 nm (n=3).

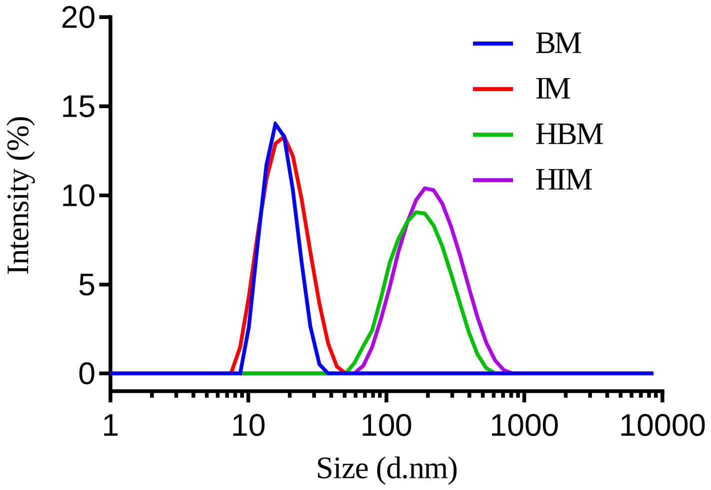

Supplementary Fig. 7. Size distribution of BM, IM, HBM and HIM were determined by

DLS.

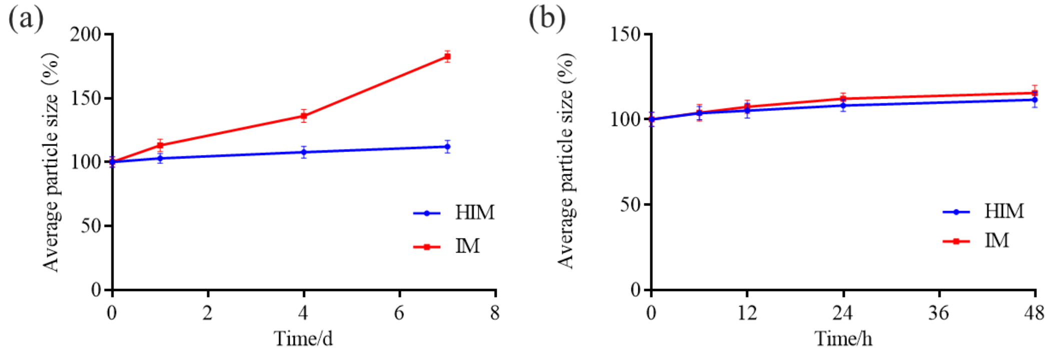

Supplementary Fig. 8. Size stability of micelles stored at (a) 4℃ and (b) 37℃ (C_{ICG}=10 μg/mL, n=3).

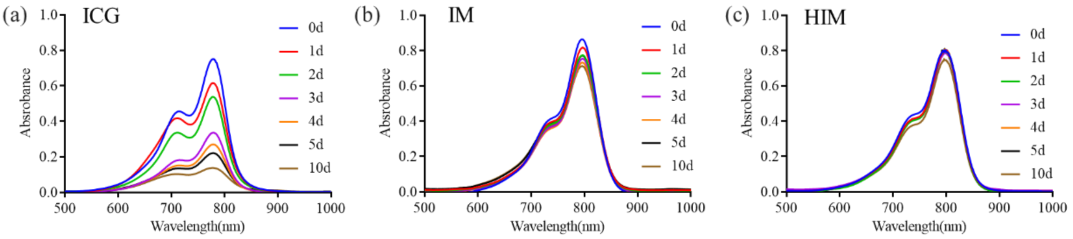

Supplementary Fig. 9. UV-vis absorption spectra of (a) ICG, (b) IM and (c) HIM within 10 days (C_{ICG}=10 μg/mL).

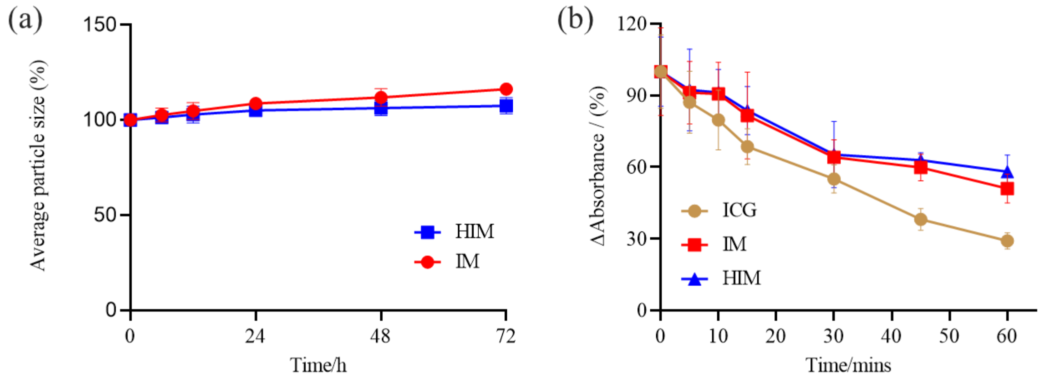

Supplementary Fig. 10. (a) Serum stability of micelle sizes and (b) photostability of ICG content reduction (initial C_{ICG}= 10 μg/mL, n=3).

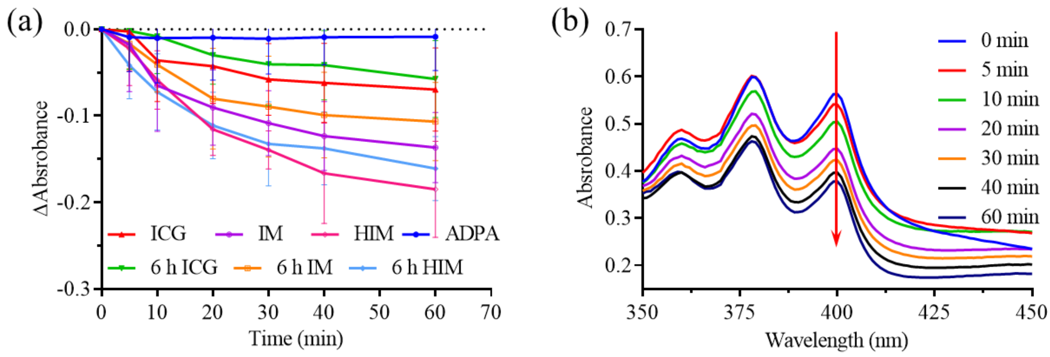

Supplementary Fig. 11. (a) △Abs of ADPA decreasing at 400 nm by irradiating samples (C_{ICG}=10 μg/mL, C_{ADPA}=20 μg/mL) with an 808 nm laser (372 J/cm^2, n=3); (b) UV-vis spectra of HIM (C_{ICG}=10 μg/mL) with 20 μmol/L ADPA exposing under 808 nm laser (0.32 W/cm^2) within 60 mins (n=3).

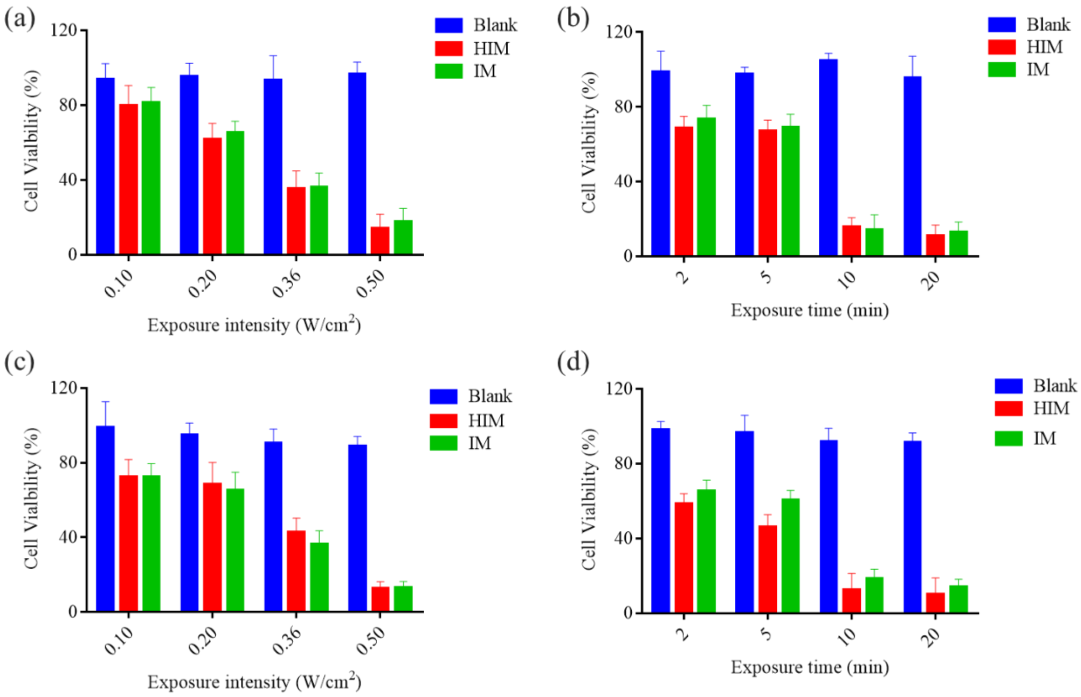

Supplementary Fig. 12. PDT effect of varied exposure intensity in 20 mins on (a) 4T1 (c) MDA-MB-231 cells, PDT effect of varied exposure time (0.62 W/cm^2) on (b) 4T1 (d) MDA-MB-231 cells. (**$p < 0.01$ comparing to blank, C_{ICG}=10 μg/mL, n=6).

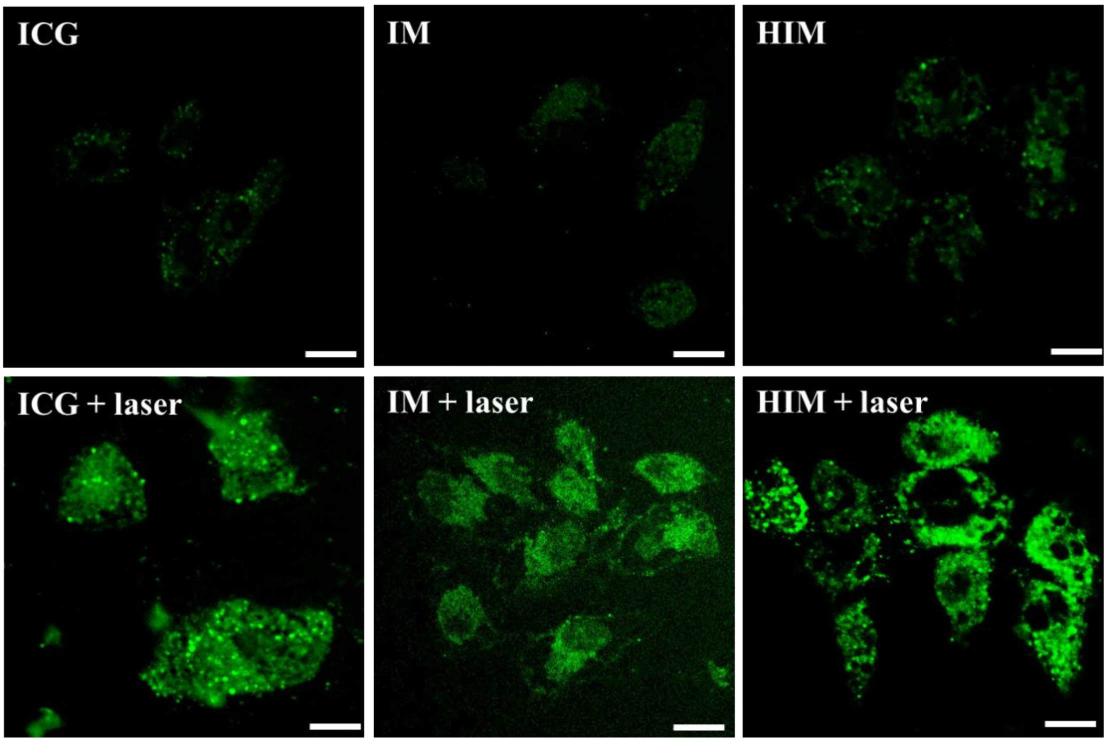

Supplementary Fig. 13. Intracellular reactive oxygen species (ROS) generation was treated with ICG, IM and HIM irradiating with an 808 nm laser (372 J/cm^2). Green fluorescence under two photon confocal microscopy indicated positive staining for ROS probe 2',7'-Dichlorodihydrofluorescein diacetate, DCFH-DA (10 μmol/L, E_x=502 nm, E_m=523 nm). The concentration of ICG was 10 μg/mL. Scale bars, 20 μm.

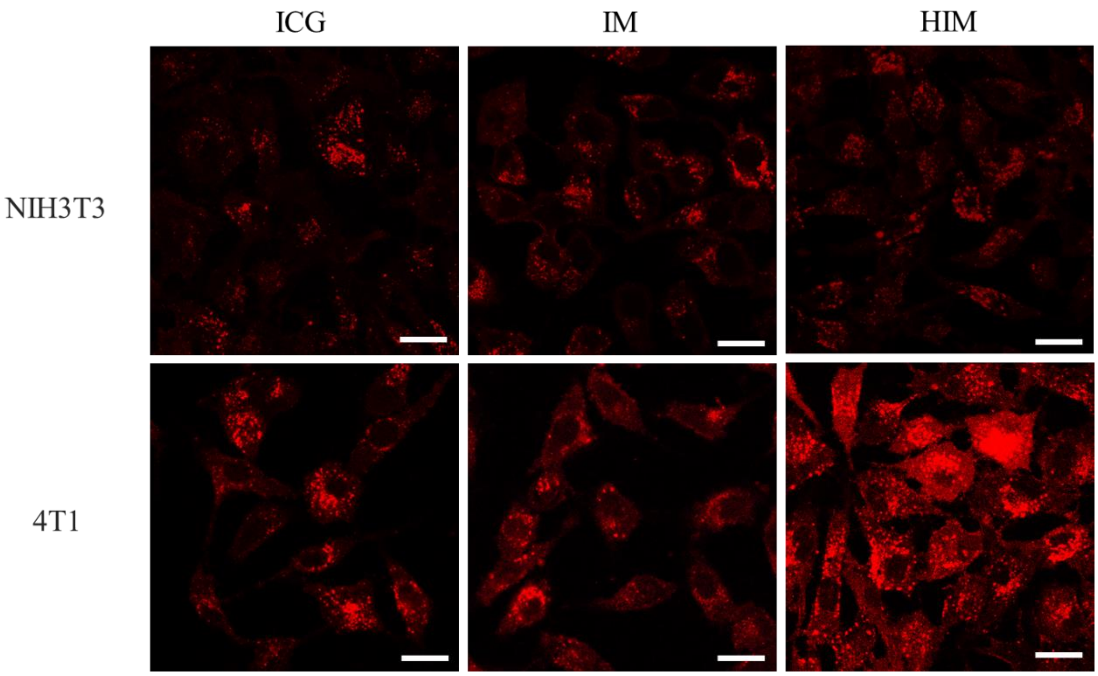

Supplementary Fig. 14. Cellular uptake of ICG, IM and HIM by NIH3T3 and 4T1 cells (C_{ICG}=10 μg/mL, E_x=780 nm, E_m=810 nm), Scale bars, 20 μm.

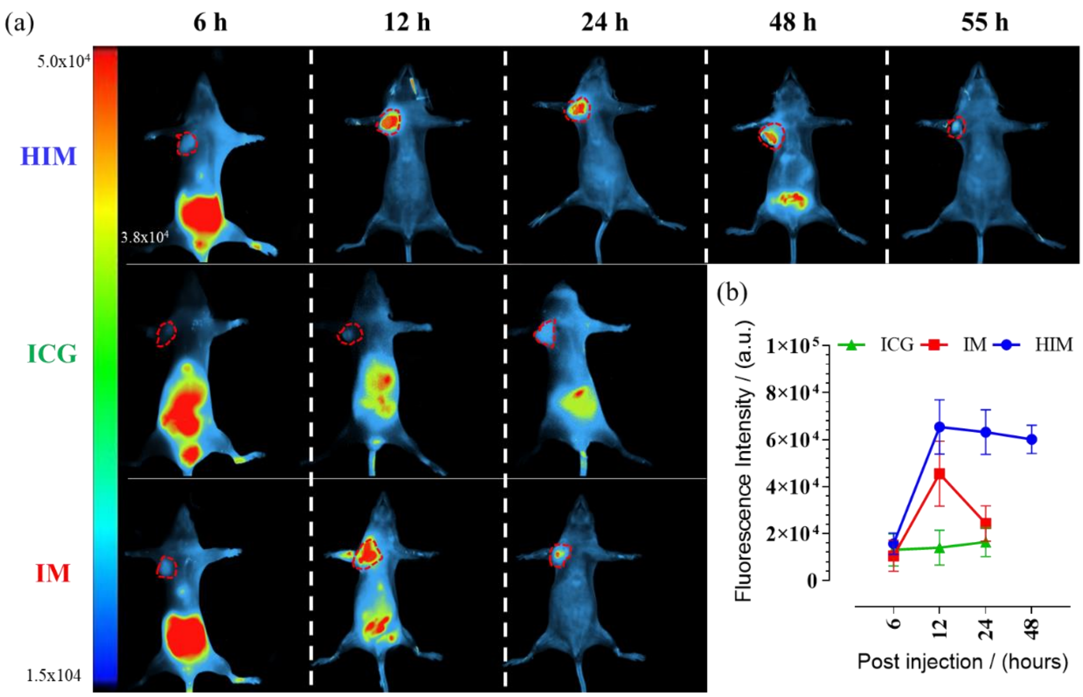

Supplementary Fig. 15. (a) Biodistribution of ICG, IM and HIM on tumor-bearing mice within 55 hours post i.v. injection (20 μg/mL, E_x=780 nm, E_m=810 nm); (b) Quantification of average fluorescence intensity in tumor (n=3).

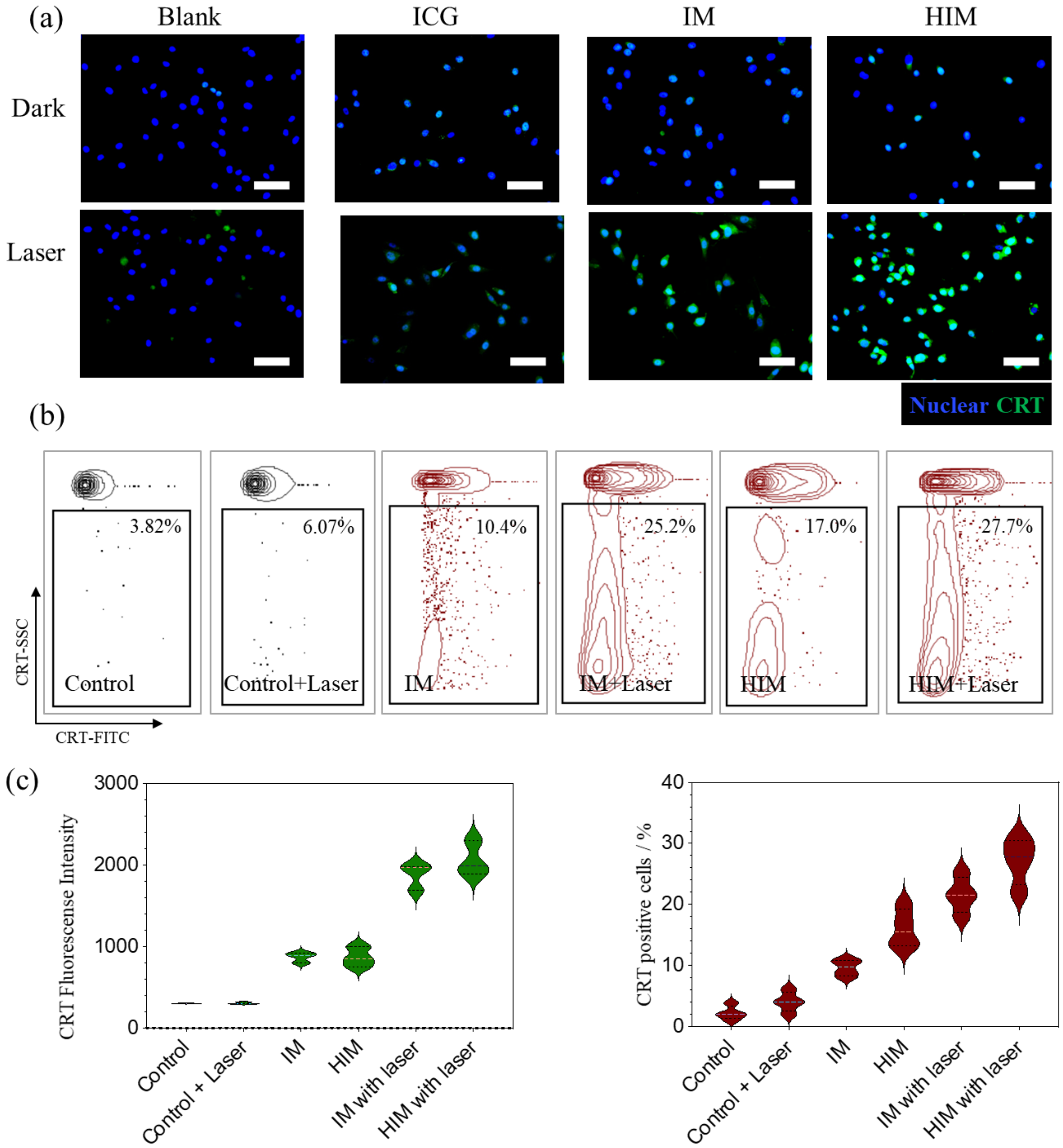

Supplementary Fig. 16. The expression of CRT on 4T1 cells after laser irradiation (808 nm, 60 J/cm^2) and positive cells were determined by (a) immunofluorescence and (b) Flow cytometry (Scale bars, 50 μm, C_{ICG}=10 μg/mL). (c) Quantification analysis of immunofluorescence and Flow cytometry (n=3).

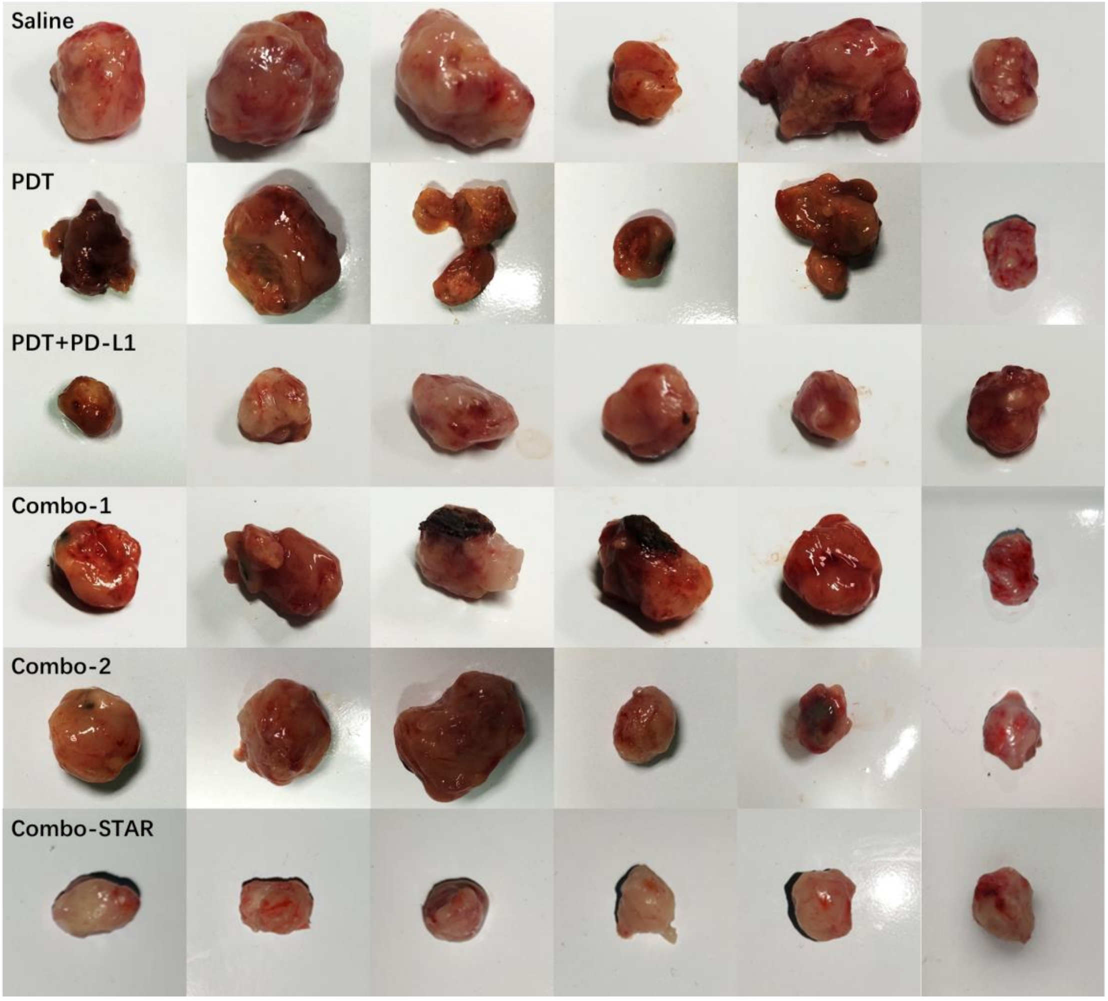

Supplementary Fig. 17. Photographs of the dissected tumor tissue from each group.

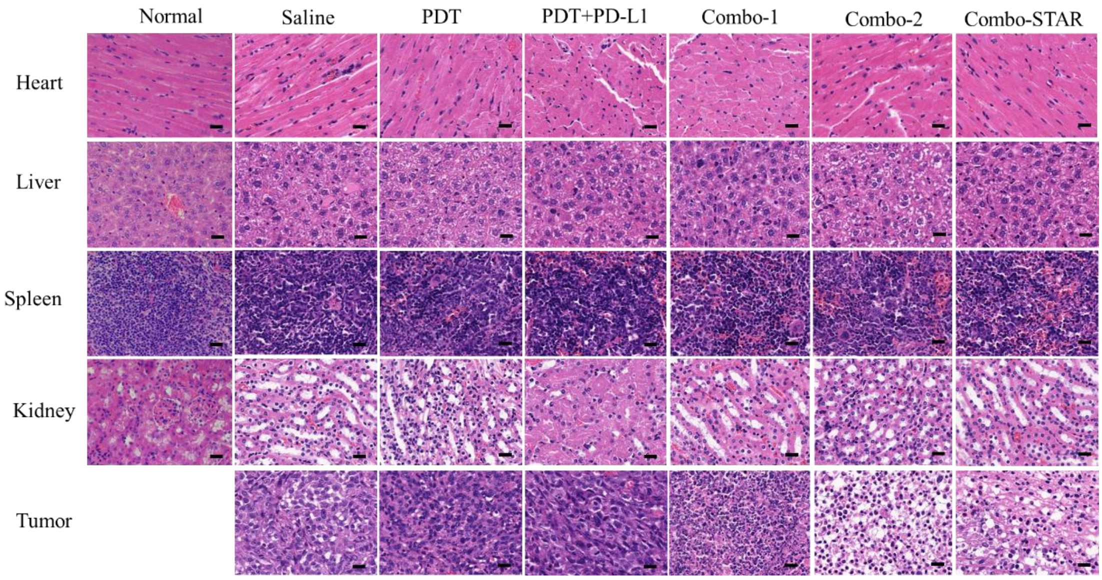

Supplementary Fig. 18. H&E staining of main organs and tumors. Scale bars, 20 μm.

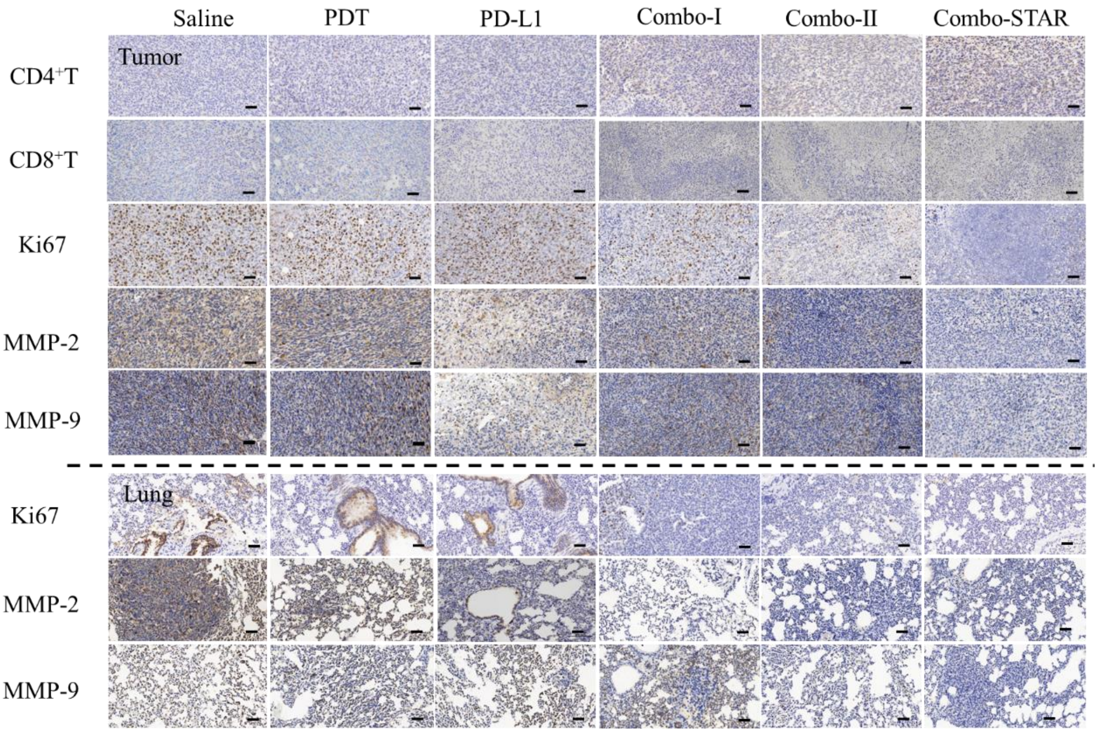

Supplementary Fig. 19. Immunohistochemical staining of tumors and lungs. Scale bars, 50 μm.

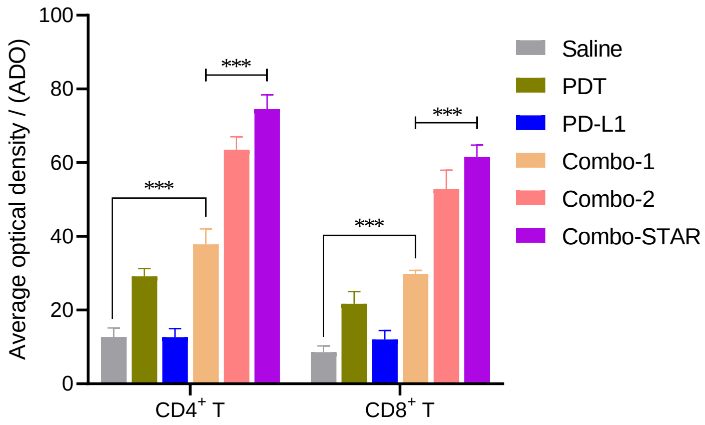

Supplementary Fig. 20. The quantitative analysis of the expression of $CD4^+$ T cells and $CD8^+$ T cells in each group (n=3). ***p<0.001.

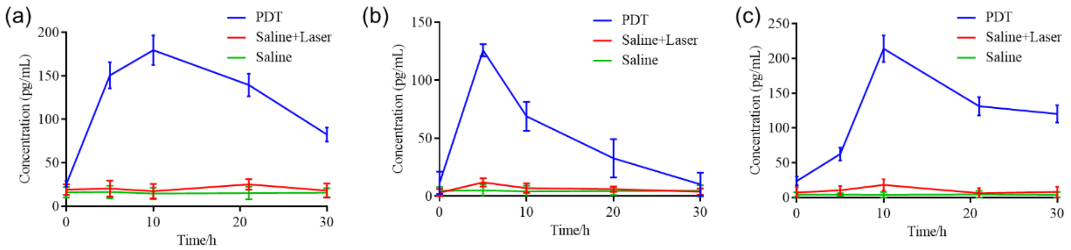

Supplementary Fig. 21. The expression of (a) IFN-γ, (b) IL-6 and (c) TNF-α after dosing with HIM (C_{ICG}=6 mg/kg, 808nm, 345.6 J/cm^2) or saline intravenously (n=3).

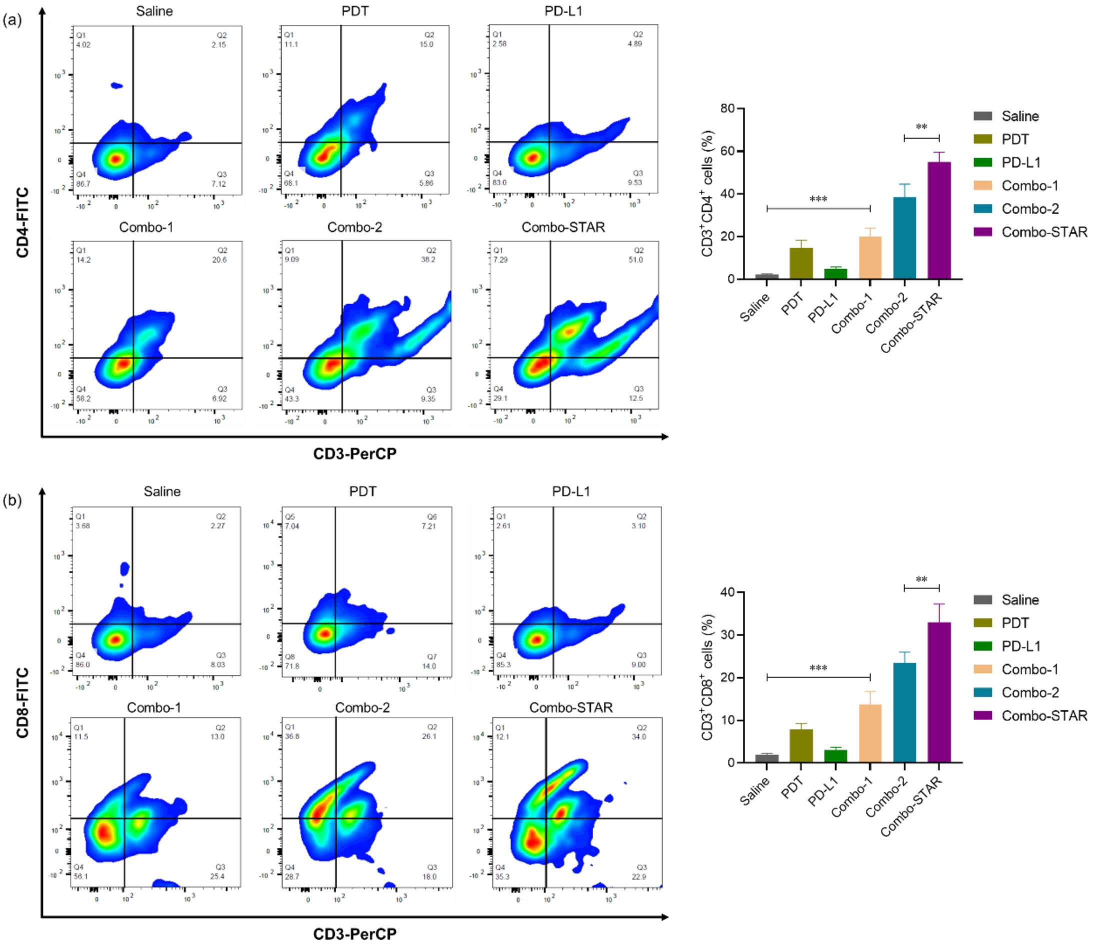

Supplementary Fig. 22. Flow cytometry analysis of the expression of (a) CD4$^+$ T lymphocytes and (b) CD8$^+$ T lymphocytes in tumors of each group (n=3). **p<0.005, ***p<0.001.

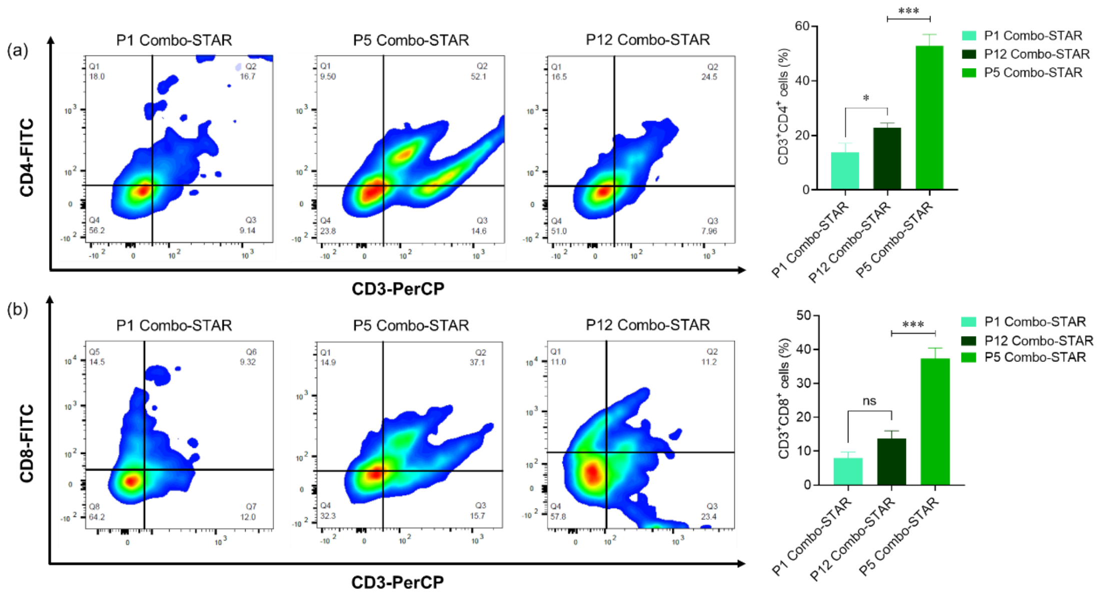

Supplementary Fig. 23. Flow cytometry analysis of the expression of (a) $CD4^+$ T lymphocytes and (b) $CD8^+$ T lymphocytes in the therapeutic process at the 1st, 5th and 12th days (n=3). *p<0.05, ***p<0.001, ns, no significance.

Supplementary Table 1. Characteristics of DLM and CLM.

Formulations	Size （DLS, nm）	Size （TEM， nm）	PDI	Zeta-Potential （mV）	E_E（%）	D_L（%）
BM	23.90 ± 3.52	19.65 ± 5.61	0.127	-20.5 ± 0.62	-	-
IM	28.91 ± 3.60	19.87 ± 5.53	0.115	-21.3 ± 0.54	66.81 ± 6.20	4.75 ± 0.60
HBM	106.40 ± 5.43	37.86 ± 5.54	0.193	-34.4 ± 1.84	-	-
HIM	136.50 ± 5.19	41.93 ± 7.86	0.141	-37.8 ± 1.21	68.92 ± 5.60	5.91 ± 0.50

Supplementary Table 2. The tumor inhibition rate of each treatment group.

Groups	Tumor inhibition rate
Combo-STAR	75.99 %
Combo-I	52.17 %
Combo-II	57.26 %
PDT	41.40 %
PDT+α-PD-L1	45.64%
